# Mindfulness Practice Is Associated With Subjective Wellbeing Homeostasis Resilience in People With Crohn's Disease but Not Ulcerative Colitis

**DOI:** 10.3389/fpsyt.2022.797701

**Published:** 2022-02-28

**Authors:** Kimina Lyall, Lauren Beswick, Subhadra Evans, Robert A. Cummins, Antonina Mikocka-Walus

**Affiliations:** ^1^School of Psychology, Deakin University, Melbourne, VIC, Australia; ^2^Barwon Health, Department of Gastroenterology, Geelong, VIC, Australia; ^3^School of Medicine, Deakin University, Geelong, VIC, Australia

**Keywords:** mindfulness, subjective wellbeing (SWB), homeostasis, resilience, inflammatory bowel disease, stress, depression

## Abstract

**Objectives:**

People with Crohn's disease and ulcerative colitis (inflammatory bowel disease: IBD), commonly experience high levels of depressive symptoms and stress and low levels of subjective wellbeing (SWB). Mindfulness is increasingly considered an adjuvant IBD treatment. The relationships between depression, disease symptoms and mindfulness have not previously been considered within the theory of SWB homeostasis. This theory states that SWB is normally maintained by a homeostatic system around a setpoint range but can fail when psychological challenges dominate consciousness. This study explored the relationship among SWB and patient-reported psychological and IBD symptoms and investigated whether mindfulness practice is independently associated with SWB homeostatic resilience.

**Design:**

This cross-sectional study recruited participants through online IBD support groups.

**Methods:**

Participants (*n* = 739; 62% Crohn's disease) detailed symptoms of depression and stress, patient-reported disease symptoms, and regularity of mindfulness practice.

**Results:**

The sample had significantly lower SWB (hedges g = −0.98) than normative data. A logistic regression found mindfulness practice doubled the Crohn's disease participants' odds of reporting SWB within the normal homeostatic range, after controlling for psychological, physical, and demographic variables (OR 2.15, 95% CI: 1.27, 3.66). A one-point increase of patient-reported bowel symptoms reduced the participant's odds of reporting SWB in the normal homeostatic range by about a third (OR 0.66, 95% CI: 0.50, 0.85). However, the influence of mindfulness or disease symptoms on SWB was not observed for people with ulcerative colitis.

**Conclusion:**

These findings provide initial evidence for an association between mindfulness and SWB homeostatic resilience in a clinical population.

## Introduction

The umbrella term “inflammatory bowel disease” (IBD) refers to two chronic, gastrointestinal conditions: ulcerative colitis (UC) and Crohn's disease (CD). Symptoms include abdominal pain, changed bowel habit, rectal bleeding, urgency and loss of appetite (1). IBD is increasing in prevalence across ethnic groups and geographies (2). Its etiology and pathogenesis are unclear, with genetic susceptivity accounting for only 20–25% of cases (3). The interaction of internal microbial factors and external environmental factors in IBD is the subject of many studies, but a clear picture is yet to emerge.

The disease's unpredictability, the uncertain nature of flares and their triggers, and its socially awkward symptoms contribute to significant psycho-social anxieties for people with IBD. These range from specific stressors relating to always needing to be near a toilet (4), to general worries such as body image or the effect of IBD on one's future (5). Consequently, the psychological impacts of living with IBD are increasingly being investigated, with depression and psychological stress key factors (6–11).

Quality of life measurements are commonly used as outcome indicators for IBD research (12–14); however, most quality of life measures focus on health outcomes and do not capture all elements of patient experience (15). An alternative measure is subjective wellbeing (SWB), which measures the subjective assessment of satisfaction with life.

A strength of SWB as a tool for psychological assessment of clinical populations is that it is grounded in theory and empirical research. One key theory is the theory of subjective wellbeing (SWB) homeostasis. The theory (16–18) builds on evidence of remarkable SWB stability across general populations, where most people indicate they are about 75% satisfied with their lives (16). The theory proposes that each person has a SWB setpoint, which is an individual difference. Maintenance of SWB within the individual's setpoint range is governed by a homeostatic system that, when operating effectively, applies cognitive and behavioral resources to maintain equilibrium (17). At population levels, setpoint ranges have been shown to be between 70 and 90 points on a 0–100% point (pp) scale (19). However, SWB homeostasis theory has not previously been applied to research on an IBD population.

According to the theory, SWB homeostasis protects an underlying, and constant, affect known as homeostatically protected mood [HPMood: (20)]. This mood is the internal state of affective equilibrium for each person when SWB is at its setpoint. This underlying internal mood state is thus the information drawn upon to answer questions about levels of satisfaction with life (18). However, stressors challenge homeostatic control, as the high levels of emotional content shifts awareness away from the underlying HPMood. If stress is strong and chronic, homeostatic control chronically fails. The consequence is a level of SWB that lies persistently outside its setpoint range. Under such conditions, negative affect dominates consciousness, with a consequential increase in the likelihood of depression (20).

This theoretical approach could be useful for studying an IBD population as there is an emerging consensus that psychological stress and depression are part of the lived experience of people with IBD (4, 5, 7, 8, 11, 21). Thus, it is possible that people with IBD with depression symptoms are experiencing SWB homeostatic defeat.

The connections among stress, depression and IBD have invited explorations into the effectiveness of mindfulness interventions for reducing psychological symptoms for people with IBD. Mindfulness is theorized to reduce stress, and possibly inflammation, through a set of techniques that target the autonomic nervous system (22). Mindfulness has been shown to support emotional regulation through positive reappraisal (23) and extinction of habitual emotional responses to stressors (24, 25) benefitting psychological symptoms such as depression and anxiety (26–30). There is, however, mixed evidence of mindfulness reducing stress in people with IBD. A systematic review found few effects (29), however, subsequent studies involving people with CD (31) and UC (32) provide some support for the role of mindfulness in reduced subjective stress. Results from a recent randomized control trial showed that a mindfulness intervention could improve IBD biomarkers (33). Another recent study found trait mindful awareness mediates the association between disease severity, quality of life, fatigue, and stress (34). In summary, evidence for the effectiveness of mindfulness in reducing psychological symptoms associated with IBD is uncertain, but emerging (29, 35).

A challenge of mindfulness research involves the conceptualization and measurement of mindfulness (36, 37). While intervention studies can be constructed to measure the impact of specific mindfulness techniques or training on outcomes of interest [e.g., (38)], cross-sectional studies have largely relied on mindfulness questionnaires that capture dispositional traits associated with mindfulness practice (39). However, these measures are agnostic to levels of mindfulness practice, and indeed whether participants engage in formal mindfulness techniques at all. An alternative approach is to conceptualize mindfulness as a health-related behavior, rather than a mental state or personality trait, and to explore whether active participation in such behavior influences outcome measures. While this approach has been adopted for research into the relationship between mindfulness practice and mindfulness measures (40), emotional reactions of cyclists (41), stress and inflammatory responsivity (42) and subjective wellbeing (43), to our knowledge no IBD studies have explored the impact of regular mindful practice on outcomes of interest.

This study responds to a growing call for more specific research into the environmental triggers, including psychological stress, that contribute to IBD (9). It aims to understand the relationship between SWB homeostasis and psychological and physical symptomology for people with IBD; and to explore whether mindfulness practice is independently associated with SWB homeostatic resilience in people with IBD, after controlling for other psychological and demographic factors. We hypothesized that:

1. Compared to normative data, an IBD sample will demonstrate lower levels of SWB.
2. Patient-reported levels of stress and depression symptoms will be negatively associated with SWB.
3. Patient-reported IBD symptoms will be negatively associated with SWB.
4. After controlling for the influence of demographics, patient-reported stress, and depression symptoms and patient-reported IBD symptoms, mindfulness practice will independently predict an enhanced probability that SWB scores will fall within the normal homeostatic range (i.e., between 70 and 90 pp).

## Methods

### Design

A cross-sectional survey was conducted, using an on-line questionnaire.

### Participants

Participants were recruited through the distribution of recruitment flyers in social media groups established for people with IBD in Australia in late 2019. Participants were asked to complete an online Qualtrics survey if they were aged over 18 and had a diagnosis of CD or UC. More than 900 participants began the survey, which took around 10 min to complete. Participants who failed to provide answers for all variables of interest were excluded, leaving *n* = 739 for analysis.

### Measures

#### Subjective Wellbeing

The Personal Wellbeing Index (PWI) (44) comprises seven items, each representing a life domain (standard of living, health, achieving in life, relationships, safety, community connection and future security). Responses involved an 11-point, end-defined scale, anchored by not satisfied at all (0) and completely satisfied (10). The composite and average of these items measures subjective wellbeing (SWB) as a continuous variable, with Cronbach's alpha = 0.89.

Total PWI scores were standardized to a 0–100% point (pp) scale (44). Cases with missing values in one or more domains were coded as missing data, and, in accordance with the manual, respondents with PWI scores of 0 (*n* = 2) or 100 (*n* = 1) were excluded. PWI total scores between 70 and 89.9 pp were categorized within normal homeostasis range.

#### Depression and Stress

Self-reported symptoms were measured using items derived from the Depression and Stress scales of the Depression, Anxiety and Stress Scales (45). To reduce the number of items in the survey, 4 items were chosen from each of the scales, following research into strongest loading factors (46). An exploratory factor analysis indicated the items loaded on to two factors as expected, with no cross-loadings >0.4, and thus the items chosen were determined to have an acceptable factor structure. The items employed were depression; I couldn't seem to feel any positive feelings at all, I felt that I had nothing to look forward to, I felt downhearted and blue, I felt I wasn't worth much as a person. For stress: I found it hard to wind down, I found myself getting agitated, I found it difficult to relax, I felt that I was rather touchy. Respondents were asked on an end-defined 11-choice scale anchored by not at all (0) to completely (10), how much the item applied over the past week. The scale was chosen following recommendations 11-point scales produce clearer normal distributions (47, 48). Cronbach's alpha was 0.83 and 0.90 for stress and depression, respectively. Cases that contained missing values in one or more items had the relevant symptom score coded as missing.

#### Disease Symptoms

IBD symptoms were measured by CD patient reported outcome signs and symptoms (49) and the UC counterpart (50), both of which were developed to respond to US Food and Drug Administration guidelines. Each measure has two scales: bowel and abdominal functioning outcomes, with higher scores indicating more frequent symptoms. Example questions are: “over the last 24 h, how many bowel movements did you have?” and “over the past 24 h, did you have pain in your belly?” For abdominal functioning, the items for both disease conditions are identical. For bowel symptoms, there are three common items across both disease conditions; and UC patients are asked an additional three items (relating to blood in bowel movements, mucus in bowel movements and leaking prior to reaching the toilet). Total scores were calculated according to the authors' instructions, with separate scores relating to patient reported bowel symptoms and patient reported abdominal symptoms. Total scores for cases with one or more missing items were coded as missing. Cronbach's alpha for bowel symptoms was 0.74 for CD and 0.80 for UC. Cronbach's alpha for abdominal symptom items was 0.58 for CD and 0.63 for UC, slightly lower than the scale validation studies. Due to its low reliability, this scale was not used in this study.

Symptom severity was categorized following cut-offs outlined in the additional files to the above papers (49, 50), with moderate or severe symptoms cut-offs as follows: CD bowel symptoms, a score of 2.4 or more; UC bowel symptoms, a score of 1.2 or more. These categories were used for descriptive statistics only, to present a picture of the clinical characteristics of the sample. Inferential analyses used the continuous variables and divided the sample into separate diagnosis cohorts.

#### Mindfulness

Participants were asked how often (never, occasionally, weekly, a couple of times a week and daily) they practice mindfulness. Mindfulness practiced was described as including focused breathing, body scans, yoga exercises, or guided meditations. Responses were coded as “yes” or “no”. To be conservative, answers of “never” or “hardly ever” were categorized as no and “occasionally,” “weekly,” “a couple of times a week,” or “daily” were categorized as yes.

#### Demographics

Participants were also asked to report their gender, age, income, disease type, when they were diagnosed, and whether they have another chronic disease.

### Statistical Analyses

SPSS version 26.0 was used for analyses. All variables of interest had <3% of missing values, except for regularity of mindfulness practice (6.1%). The Little's MCAR test (chi-square = 503.65, df = 530, *p* = 0.79), indicated a pattern of data missing completely at random. Thus, subsequent analyses were conducted with pairwise deletion. Twenty-four univariate outliers (z-scores > ±3.29) were identified involving 18 cases. Correlation analyses using all cases and excluding cases containing outliers were compared. None of the differences were significant, thus, all outliers were retained.

For hypothesis 1, mean effect size comparisons with normative data from a general Australian adult population (51) were conducted. For hypotheses 2 and 3, Spearman's correlational analysis was conducted. For hypothesis 4, logistic regression employed SWB homeostasis status (scores within normal homeostasis range or not) as the outcome variable, with mindfulness practice, stress, and depression symptoms, and bowel symptoms as predictors. Analysis of the impact of gender, income, age, presence of additional chronic disease and when IBD was diagnosed was conducted using Spearman's correlations. As each of these correlated significantly with at least one patient-reported disease symptom, these were controlled for in the analysis.

### Ethical Considerations

Ethics approval was obtained from the university human research Ethics Committee.

## Results

The majority of the sample (85.6%; *n* = 625) was female; with 14.2% (*n* = 104) male and 1 participant identifying as “other gender”. Age ranged from 18 to 78, with a mean of 37.86 and standard deviation of 11.98 years. CD was the most common IBD condition (62.4%). A sizeable proportion (43%) indicated they also live with another chronic health condition. Between 30 and 51% of people with CD and 47 and 64% of people with UC self-reported disease symptoms at moderate or severe levels (Table 1).

**Table 1:** Sample's demographic and clinical characteristics.

**Variable**	**Count**	**%**
**Gender**		
Female	625	84.6
Male	104	14.1
Other	1	0.1
**Subjective wellbeing**		
<70 pp	437	59.2
70–89.9 pp	264	35.7
>90 pp	19	2.6
**IBD type**		
Crohn's	461	62.4
UC	277	37.5
**When diagnosed**		
Past month	5	0.7
Past year	38	5.1
Past 2 years	63	8.5
Past 5 years	135	18.3
5–10 years ago	183	24.8
10–20 years ago	191	25.8
>20 years ago	123	16.6
**CD bowel symptoms**		
Mild	319	69.2
Moderate or severe	139	30.2
**UC bowel symptoms**		
Mild	144	52.0
Moderate or severe	130	46.9
**Other chronic conditions**		
Yes	318	43.0
No	419	56.7
**Mindfulness practice**		
No	435	58.9
Yes	259	35.0

*IBD, inflammatory bowel disease; CD, Crohn's disease; UC, ulcerative colitis*.

The results supported hypothesis 1, that compared to normative data from a general adult population, an IBD sample will demonstrate lower SWB. Participants' mean SWB scores were significantly lower than normative data, with a mean difference of −12.4 pp (hedge's g: −0.98; 95% confidence interval: −1.06, −0.91), which indicate a difference of one standard deviation (Table 2).

**Table 2:** Means and standard deviations.

**Variable**	***n***	**Mean**	**SD**	**Norm mean^†^**	**Norm SD^†^**
Subjective wellbeing	720	62.99	16.88	75.39	12.54
Depression	730	36.01	24.69	n/a	n/a
Stress	730	49.61	22.75	n/a	n/a
Mindful practice	694	2.38	1.38	n/a	n/a
**CD patient reported symptoms**					
Bowel	459	1.92	1.03	n/a	n/a
**UC patient-reported symptoms**					
Bowel	274	1.41	0.93	n/a	n/a

*Depression and Stress measures were based on a subset of items derived from the Depression, Anxiety and Stress Scale (45)*.

† *Normative mean and standard deviations sourced from Khor et al. (51), n > 60,000*.

*CD, Crohn's disease; UC, ulcerative colitis*.

*Patient reported bowel symptoms measured with Crohn's disease/ulcerative patient reported outcomes signs and symptoms scales (49, 50)*.

Results supported the hypotheses 2 and 3, that patient-reported psychological and disease symptoms will be negatively associated with SWB. As shown by the Spearman correlational matrix (Table 3), SWB negatively correlates with depression symptoms, stress, and bowel symptoms for both the CD and UC cohorts.

**Table 3:** Spearman's correlation matrix showing relationships between psychological, disease, demographic and mindfulness characteristics.

	**SWB**	**Depression**	**Stress**	**Gender**	**Age**	**Income**	**Time since diagnosis**	**Mindful practice**	**Other chronic condition**
Depression	−0.57^***^								
Stress	−0.40^***^	0.65^***^							
Gender	0.09^*^	−0.14^***^	−0.04						
Age	−0.04	−0.03	−0.11^***^	−0.00					
Income	0.26^***^	−0.21^***^	−0.07	0.01	0.05				
Time since diagnosis	−0.01	−0.01	−0.05	−0.00	0.46^***^	0.05			
Mindful practice	0.07	−0.15^**^	−0.12^***^	−0.02	0.11^***^	−0.02	0.03		
Other chronic condition	−0.17^***^	0.06	0.06	0.01	0.16^***^	−0.03	0.07^*^	0.12^***^	
CD-Bowel	−0.31^***^	0.28^***^	0.19^***^	−0.03	0.08	0.03	0.09	−0.08	0.04
UC-Bowel	−0.29^***^	0.22^***^	0.22^***^	−0.04	−0.03	−0.21^***^	−0.03	−0.13^*^	0.05

* *Significant at 0.05*.

** *Significant at 0.01*.

*** *Significant at 0.001*. *SWB, Subjective wellbeing*. *Depression and Stress measures were based on a subset of items derived from the Depression, Anxiety and Stress Scale (45)*. *Mindful practice = self-reported regularity of practice*. *CD-Bowel = Crohn's disease Patient Reported Bowel Symptoms*. *UC-Bowel = ulcerative colitis Patient Reported Bowel Symptoms*. *Participants completed the patient-report scales relevant to their disease diagnosis, hence there are no correlation coefficients between the diagnosis categories*.

Hypothesis 4 proposed that, after controlling for the influence of demographic, patient-reported stress, depression symptoms and IBD bowel symptoms, mindfulness practice would independently predict SWB in the normal homeostasis range (i.e., scores between 70 and 90 pp). A binomial logistic regression was performed, with the data file split according to IBD-type. Age, income, gender, when diagnosed, and presence of another chronic disease were included in the model because each significantly correlated with either disease symptoms or SWB. All logistic regression assumptions were met. The dependent variable was coded such that 0 = a SWB score outside the normal homeostatic range, and 1 = a score inside of the normal homeostatic range. Results are shown in Table 4.

**Table 4:** Predictors of subjective wellbeing in the normal homeostasis range for people with CD or UC.

		**Odds of subjective wellbeing within normal homeostatic range (70–90 pp)**	
**CD**	**Predictor**	**Odds ratio**	**(95% CI)**
	Engage in mindfulness practice (if yes)^**^	2.15	(1.27, 3.66)
	Depression score higher^***^	0.97	(0.95, 0.98)
	Stress score higher^ns^	1.00	(0.98, 1.01)
	Patient-reported Bowel Symptoms higher^**^	0.65	(0.50, 0.85)
	Greater time since diagnosis^ns^	0.97	(0.79, 1.18)
	Having another chronic condition (if yes)^ns^	0.89	(0.52, 1.52)
	Gender (if female)^ns^	1.40	(0.62, 3.15)
	Older age^ns^	0.98	(0.95, 1.00)
	Higher income^*^	1.25	(1.03, 1.51)
UC	Engage in mindfulness practice (if yes)^ns^	1.51	(0.81, 2.84)
	Depression score higher^***^	0.97	(0.95, 0.98)
	Stress score higher^ns^	1.00	(0.99, 1.02)
	Patient-reported Bowel Symptoms higher^ns^	0.84	(0.58, 1.20)
	Greater time since diagnosis^ns^	0.89	(0.70, 1.13)
	Having another chronic condition (if yes)^ns^	0.54	(0.28, 1.04)
	Gender (if female)^ns^	0.79	(0.32, 1.94)
	Older age^ns^	0.99	(0.96, 1.02)
	Higher income^*^	1.30	(1.03, 1.63)

*Logistic regression coefficient*

* *p < 0.05;*

** *p < 0.01;*

*** *p < 0.001;*

ns *not significant*.

*Depression and Stress measures were based on a subset of items derived from the Depression, Anxiety, and Stress Scale (45)*.

*Patient reported bowel symptoms measured with Crohn's disease/Ulcerative patient reported outcomes signs and symptoms scales (49, 50)*.

For the CD cohort, the model was statistically significant, χ^2^ (9, *n* = 341) = 97.10, *p* < 0.001. The model explained between 25% (Cox and Snell *R*^2^) and 34% (Nagelkerke *R*^2^) of the probability of being in the normal homeostatic range. It correctly classified 73% of cases (82% of SWB outside the normal homeostatic range and 59% of SWB within). Mindfulness practitioners were more than twice as likely to report SWB within the normal homeostatic range (OR 2.15, 95% CI: 1.27, 3.66), after controlling for the influence of the other variables. Bowel symptoms significantly contributed to the model. An increase of one point on the bowel symptom measure reduced the odds of the participant reporting SWB in the normal homeostatic range by about a third (OR 65, 95% CI: 0.50, 0.83). Depression symptoms, but not stress, significantly predicted the odds of reported SWB in the normal homeostatic range, such that a one-point increase in depression scores decreased the chances of the participant reporting SWB inside the range (OR 0.97, 95% CI: 0.95, 0.98). Income was the only statistically significant demographic variable, at the 0.05 level; an increase in income slightly increased the odds of reporting SWB in the normal homeostatic range (OR 1.25, 95%CI: 1.03, 1.51). No other demographic factor contributed significantly to the model.

For the UC cohort, the model was also statistically significant, χ^2^ (9, *n* = 226) = 48.87, *p* < 0.001. The model explained between 19% (Cox and Snell *R*^2^) and 26% (Nagelkerke *R*^2^) of the probability of being in the normal homeostatic range. It correctly classified 72% of cases (81% of SWB outside normal range and 58% of SWB within). Mindfulness did not significantly predict SWB homeostasis for this cohort, nor did the patient-reported physical symptoms, nor stress. Depression significantly predicted SWB homeostasis, after controlling for the other variables (OR 0.97, 95% CI: 0.95, 0.99). Income was the only statistically significant demographic variable, at the 0.05 level; an increase in income slightly increased the odds of reporting SWB in the normal homeostatic range (OR 1.3, 95% CI: 1.03, 1.63).

## Discussion

This is the first time an IBD sample has been studied within the framework of SWB homeostasis theory. This theory adds an important understanding to our knowledge of the relationship between IBD and wellbeing because it provides a framework to understand expected homeostatic resilience (SWB scores in the setpoint range; 70–90 pp).

As predicted, the IBD sample demonstrated significantly lower SWB than normative data from the general population and reported a mean SWB below the typical range of SWB homeostasis, indicative of potential homeostatic failure. Homeostatic failure represents the point at which the homeostatic system has failed to be resilient to psychological challenges and indicates that the individual has insufficient resources to apply to their stressors (17). The negative psychological impact of IBD is well-established (11, 52).

To further explore the relationships within SWB homeostasis theory, this study sought to identify psychological and disease symptoms and their associations with levels of SWB scores within the normal homeostatic range (19, 53). Depression symptoms decreased the odds of participants reporting SWB within the range, after controlling for the impact of other psychological and physical symptoms and demographics. However, the same result was not found for stress. This result was unexpected, given the proposed theoretical role of stress in SWB homeostatic defeat, as well as the prevalence of perceived stress in people with IBD (7).

Patient-reported bowel symptoms decreased the odds of reporting SWB in the resilient range for the CD cohort, but not the UC cohort. A strength of our study was the high proportion of participants with moderate or severe levels of IBD bowel symptoms, given that most prior research has largely focused on people with IBD who are not experiencing significant mental illness or active disease symptoms (38).

However, it is worth noting that patient-reported disease symptoms do not always correlate with objective markers of disease activity (54), further pointing to the need to collect biomarkers as well as subjective measures.

The study also sought to explore whether mindfulness practice could be a healthy behavior that can associated with homeostatic resilience in the presence of psychological and IBD disease symptoms. The results were also mixed. For the CD cohort, engagement in regular mindfulness practice increased the odds of a participant reporting SWB levels consistent with the normal homeostatic range, after controlling for the influence of demographic variables and psychological and IBD symptoms. However, for the UC cohort, engaging in mindfulness practice did not increase the odds of reporting SWB in the normal homeostatic range.

The finding that income was the only significant independent demographic predictor in our model, was supported by other evidence of the protective factors of resources such as income in maintaining homeostasis (55).

### Summary

Taken together, the findings provide the following picture. Participants with IBD experience lower levels of subjective wellbeing than the healthy population, at average levels indicative of SWB homeostasis defeat. Participants who have depression symptoms are more likely to report levels of SWB outside of the homeostasis range. Additionally, people with CD who have high levels of self-reported bowel symptoms are more likely to report SWB at levels outside the homeostasis range. While we could not demonstrate a causal effect of these psychological and physical impacts, due to the cross-sectional nature of our study, it is possible that such challenges place pressure on SWB homeostasis, and this aligns with prior research and theory (20). It is possible that many individuals with IBD, particularly those with higher levels of disease symptoms, have insufficient resources to respond to homeostatic challenge, and are more likely to experience homeostatic defeat.

An important clinical question in the provision of psychological support for people with IBD involves factors or interventions that increase the chances of homeostatic resilience. In this study, engagement in regular mindfulness practice was found to be associated with SWB scores within the SWB homeostatic range, but only for the CD cohort. The interpretation of these findings could be two-fold. Either individuals with CD who engage in mindfulness practice are more likely to retain homeostatic control despite the presence of psychological and IBD symptoms; or individuals who maintain homeostatic control are more likely to engage in mindfulness practice. While either of these is feasible, the former explanation links simply to theory. This also concords with other research that indicates mindfulness is effective in mitigating stress (38) and depression (27) for people with IBD. The fact that the findings did not translate to the UC cohort is intriguing. An explanation might lie in the nature of CD as a disease, which is associated with greater levels depression and anxiety symptoms, compared to UC (52). This might result in participants deriving greater proportional benefits from psychological treatment, or a greater propensity to engage in healthy behavior. To understand these relationships further, studies that differentially analyze psychological treatment between disease conditions would be required.

SWB homeostasis theory states that the variable homeostasis is protecting is an underlying positive mood called homeostatically protected mood [HPMood: (20)]. This affect underlies all conscious experience and provides an inbuilt reference point for SWB-level management by homeostasis. The presence of HPMood is normally masked by situationally-created emotion—i.e., anger, sadness, or joy, that are connected to a specific, temporal event. Where high levels of stress and depression symptoms are involved, these emotions obscure access to HPMood, and take control of SWB levels away from the homeostatic system, leading to low levels of reported SWB (56, 57).

Linking into this explanation, mindfulness has been described as creating a kind of “mental gap” between awareness and its objects (58) or a “a ‘space' between one's perception and response” (59). It effectively holds at bay the force of the emotional content, and it has been proposed that this mental gap enables the individual to experience their underlying levels of HPMood (60). This study provides some initial, albeit preliminary, evidence to support this proposal, and aligns with a growing body of research that suggests mindfulness improves psychological outcomes by a combination of regulatory mechanisms, including cognitive reappraisal, self-regulation and emotion regulation (24, 61, 62). For people with CD who experience significant psycho-social anxieties associated with their symptoms, the ability to regulate emotional and cognitive responses to these stressors may provide an important pathway to resilient levels of SWB.

### Clinical Implications

These results indicate the potential role of SWB homeostatic resilience to mitigate the experience of psychological symptoms associated with IBD. Homeostasis theory suggests the provision of resources to individuals experiencing homeostatic defeat is essential to restore homeostasis. For an IBD cohort, this study suggests that mindfulness could potentially act as such a homeostatic resource, and this is particularly the case for people with CD. Given that once SWB returns to setpoint range, the internal stability of the homeostatic system once again resumes control, this suggests that mindfulness could be an efficient technique to assist people to manage their psychological symptoms.

A growing body of literature points to the need for gastroenterology treatment to incorporate psychological care and referrals (63), and this study endorses further research in this area. It is especially important that patients at risk of SWB homeostatic defeat are identified and provided with appropriate resources and resources alongside their medical support.

### Limitations and Future Directions

As the study was cross-sectional, it remains uncertain whether mindfulness practice could be an effective intervention for people with IBD who experience stress and depression symptoms. Prospective studies could help to test this proposition. Similarly, the direction of the relationship among psychological and physical symptoms and SWB was also unable to be determined. Future studies could be designed to better investigate the causal links between psychological symptoms, mindfulness and SWB. This study did not measure anxiety levels as it was not a construct of interest; future studies could identify whether anxiety levels predict homeostasis.

The study relied on subjective, rather than objective, measures of symptomology. Patient reports are becoming important outcome measures for clinical research (64), however, some studies have found subjective measures of patient functioning do not always correlate with objective measures of disease activity (65, 66). Future studies exploring the relationship between endoscopy or biomarkers and SWB would provide a fuller picture.

Third, this study relied on self-reports for mindfulness practice, and did not examine the discriminatory features of mindfulness practice. Not all such practices are alike, thus participants will vary significantly in the type, duration, frequency and application of mindfulness techniques, creating challenges for mindfulness measurement (37). However, to the extent that engaging in self-described mindfulness practice is protective of SWB homeostasis, these results remain relevant. Additionally, our study did not collect data on other health behaviors that could be associated with increased SWB. It is possible that people who are engaged in positive health behaviors may be more likely to undertake mindfulness practice; and this area should also be further explored in future research.

The sample was predominantly female, which is not unusual in online research with IBD populations (67–70), but future studies should seek to target more male respondents. It is possible that our study attracted people who were interested in mindfulness, however, our recruitment flyers emphasized that mindfulness experience was not required to participate in the study.

Finally, we were not able to ascertain which of the potential mindfulness mechanisms are most relevant for people with IBD. SWB homeostasis theory would suggest a role for emotion regulation in restoring psychological homeostasis, and this could be further explored in future research.

## Conclusion

This study provides some evidence that individuals with IBD are at risk of being unable to retain SWB homeostasis and indicates that this population requires specific support to shore up SWB resilience in the light of expected disease-related physical and psychological challenges. This study provides preliminary evidence that mindfulness could support SWB homeostasis, particularly for people with CD. While causal interpretations are not possible, they accord with homeostatic theory, which suggests the ability to restore homeostasis requires a reduction of the dominance of emotion in consciousness. Formal mindfulness practice has been shown to support emotional regulation (23–25). It follows that regular mindfulness practice could provide a protective factor against homeostatic defeat experienced by some people with IBD, as potentially indicated by the results in this study.

## Data Availability Statement

The raw data supporting the conclusions of this article will be made available by the authors, without undue reservation.

## Ethics Statement

The studies involving human participants were reviewed and approved by Deakin University Human Ethics Advisory Group (HEAG-HH 66_2019). The patients/participants provided their written informed consent to participate in this study.

## Author Contributions

KL contributed conceptualization, methodology, data curation, analysis, writing-original draft, and writing-review and editing. LB contributed conceptualization, methodology, and writing-review and editing. SE, RC, and AM-W contributed conceptualization, methodology, resources, writing-review and editing, and supervision. All authors contributed to the article and approved the submitted version.

## Conflict of Interest

The authors declare that the research was conducted in the absence of any commercial or financial relationships that could be construed as a potential conflict of interest.

## Publisher's Note

All claims expressed in this article are solely those of the authors and do not necessarily represent those of their affiliated organizations, or those of the publisher, the editors and the reviewers. Any product that may be evaluated in this article, or claim that may be made by its manufacturer, is not guaranteed or endorsed by the publisher.
